# Association of oxidative balance score with all-cause and cardiovascular mortality among patients with cardio-renal-metabolic disease

**DOI:** 10.3389/fnut.2025.1618184

**Published:** 2025-06-25

**Authors:** Yucui Lin, Yunxia Wang, Cailing Liu, Danjie Ye, Ziran Huang, Yangbin Ou, Wenjun Gu, Jianhong Ma

**Affiliations:** ^1^Department of Laboratory Medicine, Heyuan People’s Hospital, Guangdong Provincial People’s Hospital, Heyuan Hospital, Heyuan, China; ^2^Department of Pharmacy, Huizhou Third People’s Hospital, Guangzhou Medical University, Huizhou, China

**Keywords:** cardio-renal-metabolic, oxidative balance score, all-cause mortality, cardiovascular mortality, NHANES

## Abstract

**Background:**

Cardio-renal-metabolic (CRM) conditions are increasingly recognized as a major public health challenge, with oxidative stress playing a pivotal role in poor prognosis. The oxidative balance score (OBS) is used to assess the body’s oxidative stress status, but its link to all-cause and cardiovascular mortality in CRM patients remains unclear.

**Methods:**

We used data from participants (≥ 20 years old) in the National Health and Nutrition Examination Survey (NHANES) from 1999 to 2018. The patients were divided into four groups based on OBS using the weighted quartiles method. The relationship between OBS and both all-cause and cardiovascular mortality in CRM patients was assessed using multivariable Cox regression and restricted cubic spline (RCS) models. The differences in cumulative survival between groups were examined using Kaplan–Meier analysis and log-rank tests. Sensitivity analysis and subgroup analysis were also performed.

**Results:**

During a median follow-up of 7.9 years, there were 3,838 (25.2%) and 1,412 (8.9%) patients who died from all-cause and cardiovascular mortality, respectively. After adjusting for potential confounders, elevated OBS level was negatively related to the risk of all-cause mortality [Q2, Q3, Q4: adjusted hazard ratio (aHR) (95 confidence interval (CI%)) = 0.85 (0.75–0.96), 0.87 (0.77–0.98), 0.74 (0.62–0.88), respectively; P for trend<0.001]. Moreover, Higher OBS quartiles were linked to a decreased risk of cardiovascular mortality, while no significant reduction was observed in the lower quartiles [model 3: Q2, Q3, Q4: aHR (95CI%) = 0.96(0.77–1.19), 0.78 (0.63–0.97), 0.70 (0.53–0.93), respectively; P for trend = 0.003]. Kaplan–Meier survival analysis also indicated that patients in the highest quartile of OBS had the lowest risk of both all-cause mortality and cardiovascular mortality (log-rank test *p* < 0.001). Furthermore, restricted cubic spline analyses revealed an inverse relationship between OBS levels and the risk of both all-cause and cardiovascular death. The sensitivity analyses confirmed the stability of our findings.

**Conclusion:**

Elevated levels of OBS were negatively related to the risk of all-cause and cardiovascular mortality among CRM patients, which may offer valuable information on the role of oxidative stress status for risk stratification of mortality in CRM patients.

## Introduction

Cardiac, renal, and metabolic (CRM) disorders collectively represent a major contributor to illness and death in the United States, accounting for nearly one-third of current mortality cases (1). The functions of the cardiovascular, renal, and metabolic systems are highly interconnected (2), where deterioration in one organ system can initiate and exacerbate problems in the others, ultimately resulting in significantly higher mortality risk (3–5). However, there are fewer studies on the early identification of indicators of mortality in CRM patients.

Oxidative stress, a key pathophysiological mechanism for CRM patients, arises from an imbalance between the production of reactive oxygen species (ROS) and the body’s antioxidant defenses (6, 7). This imbalance contributes to cellular damage, chronic inflammation, and endothelial dysfunction, further exacerbating the progression of cardiovascular and kidney diseases (8). As oxidative stress is affected by numerous determinants, reliance on a single indicator does not provide a comprehensive assessment of oxidative homeostasis (9). The Oxidative Balance Score (OBS) serves as an aggregate indicator that quantifies the overall oxidative stress burden by integrating both pro-oxidant and antioxidant factors, including dietary intake, lifestyle behaviors, and environmental exposures. This score provides a more comprehensive reflection of an individual’s oxidative/antioxidant status (10). Previous studies have also shown that OBS is strongly associated with the mortality risk from various chronic diseases (11, 12).

Although accumulating evidence underscores the role of oxidative stress in the pathogenesis of cardiovascular, renal, and metabolic disorders, the relationship between composite indices of oxidative stress, such as the OBS, and mortality outcomes in patients with CRM conditions remains inadequately characterized. Prior investigations have predominantly focused on individual oxidative biomarkers or general populations, which may not fully capture the intricate oxidative inflammatory interactions present in individuals with overlapping CRM pathologies. Furthermore, the mechanistic pathways through which oxidative balance influences mortality risk in this high-risk population are still insufficiently elucidated. It is well established that oxidative stress can impair insulin signaling, compromise endothelial function, and exacerbate chronic low-grade inflammation, all of which are central to CRM pathophysiology (6–8). Additionally, emerging evidence has highlighted that oxidative imbalance correlates with the severity of metabolic syndrome and diminished antioxidant defenses, particularly in older adults, thereby suggesting a plausible mechanism by which redox dysregulation may drive disease progression and adverse outcomes (13). These considerations collectively underscore the need for further research examining the prognostic value of integrative oxidative stress metrics such as OBS in CRM populations.

Consequently, this study aims to investigate the association between OBS and the risk of all-cause and cardiovascular mortality among CRM patients. By elucidating this relationship, our findings may provide valuable insights into the importance of oxidative balance in improving long-term prognosis for this high-risk population.

## Method

### Study design and study population

Data were employed from the National Health and Nutrition Examination Survey (NHANES) database, a program administered by the Centers for Disease Control and Prevention and the National Center for Health Statistics in the US. The National Center for Health Statistics Ethics Review Board approved the study, which was conducted with the explicit written consent of all participants.

CRM disease is a constellation of conditions that includes cardiovascular disease (CVD), chronic kidney disease (CKD), and diabetes mellitus (DM) (14), and patients with CRM disease during 1999–2018 were included in this research. Participants were excluded if they met any of the following conditions: (1) age under 20; (2) presence of malignant tumors; (3) pregnancy; (4) missing OBS data; or (5) missing information on mortality or survival time. Finally, 12,886 CRM patients were included in the final analysis (Supplementary Figure 1).

### Assessment of OBS

OBS is calculated using earlier established research (15, 16). It is calculated using data on 16 nutrient intakes obtained from the initial in-person dietary recall, along with four lifestyle-related variables. These include a total of 5 pro-oxidants and 15 antioxidants, selected based on established links between oxidative stress and these factors (17). OBS elements are grouped into four categories: (1) dietary antioxidants—such as fiber, *β*-carotene, riboflavin, niacin, vitamin B6, folate, vitamin B12, vitamin C, vitamin E, calcium, magnesium, zinc, copper, and selenium; (2) dietary pro-oxidants—namely total fat and iron; (3) lifestyle antioxidants—including physical activity; and (4) lifestyle pro-oxidants—including alcohol consumption, smoking, and body mass index (BMI). All variables were equally weighted in the final OBS calculation.

Supplementary Table 1 outlines the point allocation for the OBS components. For alcohol intake, points were assigned as follows: 2 points for non-drinkers, 1 point for moderate drinkers (0 to 15 g/d for females and 0 to 30 g/d for males), and 0 points for heavy drinkers (≥15 g/d for females and ≥30 g/d for males). Other components were divided into sex-specific tertiles. Antioxidant components were given 0 to 2 points across the tertiles from lowest to highest, while pro-oxidant components were scored in the reverse order, with 0 points for the highest tertile and 2 points for the lowest tertile.

### Assessment of mortality

We used the NHANES Public-Use Linked Mortality File through December 2019,^1^ which was linked to the National Death Index (NDI) data using a probabilistic matching algorithm to determine mortality status. The cause-specific mortality data in the NDI have been shown to accurately classify deaths, with only a relatively slight possibility of misclassification. The underlying cause of death was identified according to the International Classification of Diseases, and cardiovascular mortality was defined as death due to heart diseases (codes I00–I09, I11, I13, I20–I51) and cerebrovascular diseases (codes I60-I69).

### Definition of variable

Baseline information on demographics (age, sex, race, and education), lifestyle factors (smoking, alcohol drinking, and BMI), medical conditions (diabetes, hypertension, CVD, CKD), and medication usage (glucose-lowering drugs, antihypertensive drugs, and lipid-lowering drugs) was collected through structured interviews administered by trained personnel following standardized NHANES protocols. Lifestyle factors and some medical history elements were assessed through these structured interviews, while objective clinical and laboratory data were incorporated to validate and supplement the definitions of chronic conditions wherever possible (e.g., diabetes defined using HbA1c or fasting glucose, and CKD defined by eGFR or uACR). Anthropometric measurements [height, weight, BMI, systolic blood pressure (SBP), and diastolic blood pressure (DBP)] are performed by an experienced physician. Blood samples were taken by the Mobile Examination Centers during the medical examination component of the NHANES survey, and laboratory indices were selected. Demographic data were further classified as alcohol drinking (heavy moderate; none) and smoking (current smoker; former smoker; never smoker). The eGFR was calculated using the Chronic Kidney Disease Epidemiology Collaboration (CKD-EPI) equation (18), and CKD was defined as eGFR ≤ 60 mL/min/1.73 m2 or uACR ≥30 mg/g or self-reported diagnosis history. Diabetes was defined as fasting glucose ≥ 7.0 mmol/L or Hemoglobin A1c (HbA1c) (%) ≥ 6.5 or self-reported diagnosis history of diabetes or use of any hypoglycemic medication (19). CVD was defined as being informed by a health professional of having congestive heart failure, angina, coronary heart disease, heart attack, or stroke.

### Statistical analysis

Mean ± standard error (SE) was used to describe continuous variables, with ANOVA applied for group comparisons. Categorical variables were expressed as frequencies and proportions, analyzed using the chi-square test. The patients were divided into four groups based on OBS using the weighted quartiles method. Adjusted hazard ratios (aHR) and 95% confidence intervals (CIs) for all-cause and cardiovascular mortality were estimated through multivariate Cox regression. Kaplan–Meier curves were used for survival analysis, and differences between OBS groups were assessed using a stratified log-rank test. Model 1 adjusted for age, gender, and race. Model 2 was adjusted for age, gender, race, alcohol drinking, smoking, and body mass index (BMI). Model 3 extended Model 2 by additionally adjusting for DM, CVD, chronic kidney disease (CKD), uric acid, blood urea nitrogen, HbA1c, eGFR, hemoglobin, aspartate aminotransferase (AST), systemic immune-inflammation index (SII), high-density lipoprotein cholesterol (HDL-C), total cholesterol, lipid-lowering drug, antihypertensive drug, and glucose-lowering drug. To examine the dose–response relationships between OBS and mortality in CRM patients, restricted cubic spline regression analysis was performed, and the analysis was adjusted for the variables in Model 3. Stratified analyses were performed to assess the associations between quartiles of OBS levels and all-cause as well as cardiovascular death, stratified by age, and the presence of CVD, CKD, and DM. To further investigate the potential heterogeneity of the relationship between OBS and mortality outcomes in CRM patients, we performed additional subgroup analyses based on the number of CRM conditions (1, 2, or 3). Participants who experienced mortality within the first 2 years of follow-up were excluded to minimize potential bias from reverse causation.

NHANES survey weights were applied in all analyses to accommodate its complex, stratified cluster sampling design and to produce estimates representative of the U. S. Population. R software (v4.2.1) was used for statistical analysis, and significance was defined as a two-tailed *p*-value less than 0.05.

## Result

### Population characteristics

A total of 12,886 participants from NHANES were included in our analysis. Among the 12,886 included participants, 8,599 (66.7%) had only one CRM condition, 3,377 (26.2%) had two CRM conditions, 910 (7.1%) had all three conditions (CVD, CKD, and DM) (Supplementary Figure 2). Participants averaged 57.37 ± 0.25 years in age, with females comprising nearly 52.66%. Participants were categorized into four groups according to OBS quartile. Patients with reduced OBS levels were more likely to be smokers and to have a higher prevalence of CVD and hypertension. They show lower levels of eGFR, albumin, HDL-C, but had higher levels of total cholesterol, SII. Moreover, the incidence of all-cause and cardiovascular mortality was higher among patients with lower OBS levels (Table 1).

**Table 1:** Baseline characteristics of the study population according to OBS quartile.

Variable	Total	Q1	Q2	Q3	Q4	*p*-value
	12,886	3,754 (25.7%)	3,458 (25.6%)	3,114 (25.7%)	2,560 (23.1%)	
Age, years	57.37 ± 0.25	58.20 ± 0.43	58.54 ± 0.43	57.18 ± 0.41	55.35 ± 0.49	< 0.001
Gender, *n* (%)						0.947
Female	6,491 (52.66)	1824 (53.00)	1733 (53.05)	1,624 (52.26)	1,310 (52.28)	
Male	6,395 (47.34)	1930 (47.00)	1725 (46.95)	1,490 (47.74)	1,250 (47.72)	
Race, *n* (%)						< 0.001
Hispanic	3,388 (13.88)	877 (12.93)	961 (14.72)	851 (13.98)	699 (13.87)	
Non-Hispanic black	3,287 (14.81)	1,280 (21.42)	855 (15.57)	679 (11.96)	473 (9.78)	
Non-Hispanic white	5,219 (63.75)	1,363 (58.22)	1,383 (61.96)	1,332 (66.97)	1,141 (68.31)	
Other	992 (7.56)	234 (7.42)	259 (7.75)	252 (7.09)	247 (8.04)	
Smoke, *n* (%)						< 0.001
Current	2,482 (20.66)	944 (28.88)	652 (20.64)	544 (19.27)	342 (13.14)	
Former	4,074 (30.91)	1,149 (28.91)	1,134 (31.03)	993 (31.83)	798 (32.07)	
Never	6,318 (48.36)	1,656 (42.21)	1,671 (48.32)	1,575 (48.90)	1,416 (54.79)	
Alcohol drinking, *n* (%)						< 0.001
Heavy	1,269 (11.46)	320 (10.19)	343 (11.51)	336 (13.19)	270 (10.90)	
Moderate	1,019 (8.31)	235 (5.94)	248 (7.75)	274 (9.05)	262 (10.75)	
None	10,598 (80.22)	3,199 (83.86)	2,867 (80.74)	2,504 (77.76)	2028 (78.35)	
BMI, kg.m^2^	30.97 ± 0.12	31.45 ± 0.22	31.04 ± 0.16	31.09 ± 0.22	30.22 ± 0.20	< 0.001
SBP, mmHg	130.27 ± 0.28	131.76 ± 0.49	131.50 ± 0.51	129.76 ± 0.58	127.89 ± 0.65	< 0.001
DBP, mmHg	71.25 ± 0.24	71.53 ± 0.41	70.87 ± 0.38	71.10 ± 0.40	71.53 ± 0.38	0.470
Laboratory indexes						
HbA1c, %	6.36 ± 0.02	6.35 ± 0.04	6.44 ± 0.03	6.34 ± 0.04	6.32 ± 0.05	0.095
eGFR, mL/min/1.73m^2^	85.38 ± 0.39	82.07 ± 0.73	84.63 ± 0.71	86.11 ± 0.58	88.98 ± 0.78	< 0.001
Uric acid BMI, umol/L	5.74 ± 0.02	5.93 ± 0.04	5.73 ± 0.04	5.74 ± 0.05	5.55 ± 0.05	< 0.001
BUN, mmol/L	15.67 ± 0.09	15.43 ± 0.17	15.77 ± 0.18	15.80 ± 0.17	15.70 ± 0.17	0.369
HGB, g/dL	14.09 ± 0.03	13.94 ± 0.06	14.09 ± 0.05	14.17 ± 0.04	14.16 ± 0.04	0.002
Ast, U/L	26.22 ± 0.22	26.34 ± 0.54	26.15 ± 0.50	26.01 ± 0.29	26.39 ± 0.47	0.905
Albumin, g/L	41.89 ± 0.06	41.40 ± 0.11	41.79 ± 0.09	42.11 ± 0.09	42.28 ± 0.11	< 0.001
SII	599.77 ± 6.04	625.90 ± 14.15	606.84 ± 10.90	583.85 ± 10.20	581.05 ± 9.40	0.018
Total cholesterol, mmol/L	194.23 ± 0.68	196.40 ± 1.34	193.91 ± 1.24	193.75 ± 1.23	192.78 ± 1.57	0.304
HDL-C, mmol/L	50.24 ± 0.26	49.41 ± 0.46	49.87 ± 0.40	50.19 ± 0.42	51.61 ± 0.51	0.005
Medical history, *n* (%)						
DM	6,843 (49.15)	1939 (48.49)	1908 (52.78)	1,662 (48.50)	1,334 (46.58)	0.006
CKD	6,868 (51.40)	2,121 (54.41)	1831 (50.37)	1,638 (50.83)	1,278 (49.81)	0.077
CVD	4,372 (33.88)	1,473 (37.87)	1,200 (35.49)	960 (32.41)	739 (29.28)	< 0.001
Hypertension	8,818 (64.06)	2,739 (67.85)	2,372 (65.20)	2,115 (64.13)	1,592 (58.55)	< 0.001
Drug history, *n* (%)						
Glucose-lowering drug	4,432 (31.39)	1,218 (29.19)	1,243 (34.32)	1,122 (32.79)	849 (29.19)	0.002
Antihypertensive drug	7,827 (57.13)	2,355 (57.95)	2,135 (59.81)	1908 (57.69)	1,429 (52.91)	0.003
Lipid-lowering drug	4,805 (37.63)	1,392 (37.12)	1,290 (37.96)	1,170 (37.52)	953 (38.14)	0.926
Endpoint, *n* (%)						
All-cause mortality	3,838 (25.19)	1,368 (32.24)	1,062 (26.71)	857 (23.30)	551 (17.75)	< 0.001
Cardiovascular mortality	1,412 (8.93)	519 (11.66)	410 (10.45)	292 (7.37)	191 (5.94)	< 0.001

BMI, body mass index; SBP, Systolic Blood Pressure; DBP, Diastolic Blood Pressure; HbA1c, Hemoglobin A1c; BUN, Blood Urea Nitrogen; HGB, Hemoglobin; Ast, Aspartate Aminotransferase; SII, systemic immune- inflammation index; HDL-C, High-Density Lipoprotein Cholesterol; DM, Diabetes Mellitus; CKD, Chronic Kidney Disease; CVD, Cardiovascular Disease.

### The association of OBS level with all-cause and cardiovascular mortality among CRM patients

**Figure 1:**
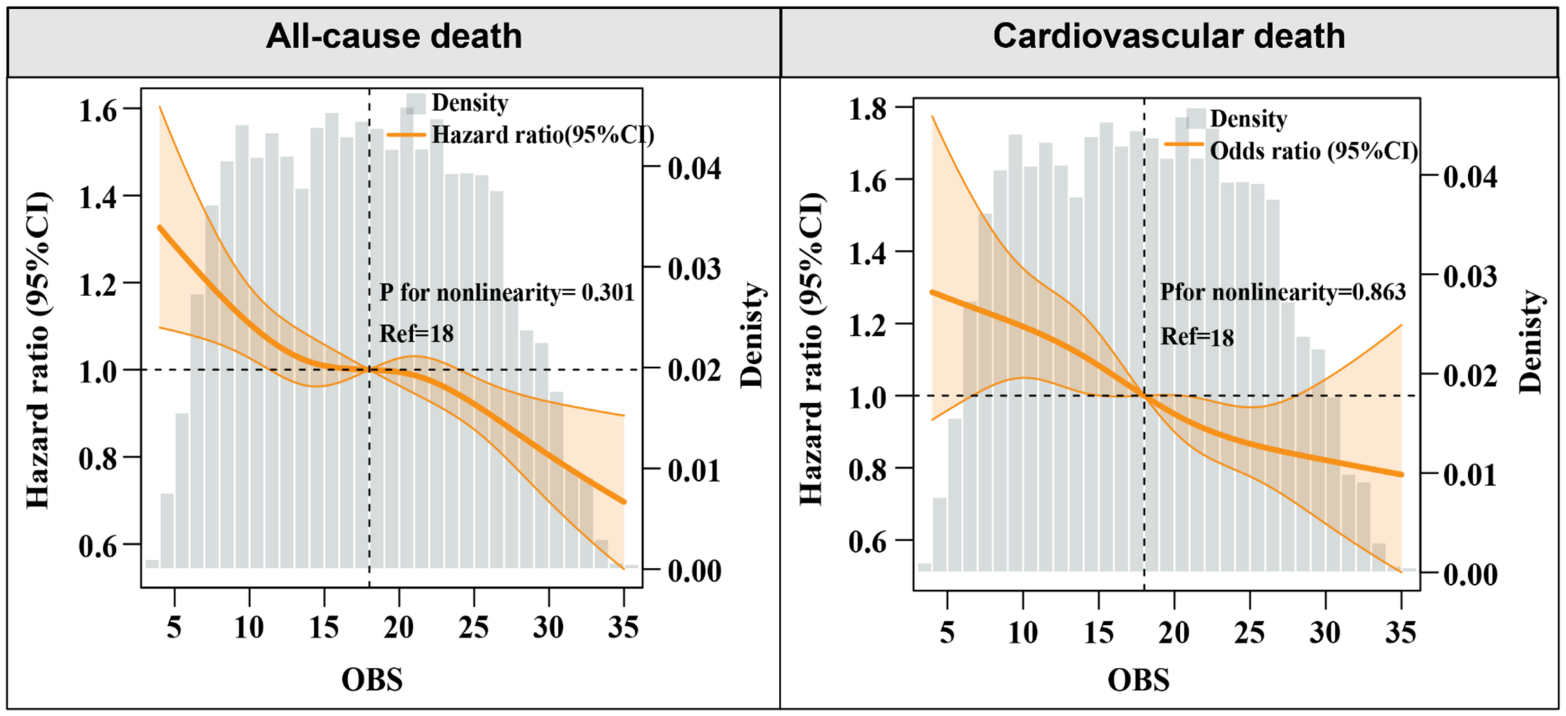
Kaplan–Meier analysis of all-cause and cardiovascular mortality according to different OBS levels.

Over a median follow-up of 7.9 years (interquartile range: 4.0–12.5 years), a total of 3,838 deaths from all causes and 1,412 cardiovascular deaths were recorded. Multivariate analyses using various models were performed to estimate the aHR for all-cause and cardiovascular mortality, considering OBS as both a continuous and categorical variable. When analyzed as a continuous variable, elevated OBS levels were independently associated with reduced risks of all-cause and cardiovascular death after adjustment for confounders in Model 2 [all-cause mortality: adjusted hazard ratio (aHR), 0.98, 95% CI: 0.97–0.99, *p* < 0.001; cardiovascular mortality: aHR, 0.98, 95%CI: 0.97–0.99, *p* = 0.003] (Table 2). In addition, restricted cubic splines also indicated a linear and negative correlation between OBS and all-cause mortality (P for nonlinear = 0.301) and CVD mortality (P for nonlinear = 0.863) (Figure 1).

**Table 2:** Univariable and multivariable cox regression analysis of the relationship between OBS levels and mortality in CRM patients.

Groups	Model 1^a^	*p*-value	Model 2^b^	*p*-value	Model 3^c^	*p*-value
	HR (95%CI)		HR (95%CI)		HR (95%CI)	
All-cause mortality						
Continuous						
OBS	0.97 (0.96,0.98)	<0.001	0.98 (0.97,0.99)	<0.001	0.98 (0.97,0.99)	<0.001
Quartiles						
Q1	1.00 (reference)		1.00 (reference)		1.00 (reference)	
Q2	0.79 (0.71,0.88)	<0.001	0.83 (0.74,0.93)	0.001	0.85 (0.75,0.96)	0.010
Q3	0.75 (0.68,0.83)	<0.001	0.80 (0.72,0.90)	<0.001	0.87 (0.77,0.98)	0.022
Q4	0.62 (0.53,0.72)	<0.001	0.70 (0.60,0.82)	<0.001	0.74 (0.62,0.88)	<0.001
P for trend	<0.001		<0.001		<0.001	
Cardiovascular mortality						
Continuous						
OBS	0.97 (0.96,0.98)	<0.001	0.97 (0.96,0.98)	<0.001	0.98 (0.97,0.99)	0.003
Quartiles						
Q1	1.00 (reference)		1.00 (reference)		1.00 (reference)	
Q2	0.85 (0.70,1.03)	0.094	0.88 (0.72,1.08)	0.245	0.96 (0.77,1.19)	0.694
Q3	0.66 (0.55,0.80)	<0.001	0.71 (0.58,0.86)	<0.001	0.78 (0.63,0.97)	0.026
Q4	0.58 (0.46,0.74)	<0.001	0.64 (0.50,0.82)	<0.001	0.70 (0.53,0.93)	0.013
P for trend	<0.001		<0.001		0.003	

a Model 1: adjusted for age, gender.

b Model 2: adjusted for age, gender, race, body mass index, smoke, alcohol drinking.

c Model 3: adjusted for age, gender, race, body mass index, smoke, alcohol drinking, hypertension, diabetes mellitus; chronic kidney disease; cardiovascular disease, hemoglobin A1c, estimated glomerular filtration rate; uric acid, blood urea nitrogen; hemoglobin; aspartate aminotransferase; systemic immune- inflammation index; total cholesterol, high-density lipoprotein cholesterol; antihypertensive drug; lipid-lowering drug.

**Figure 2:**
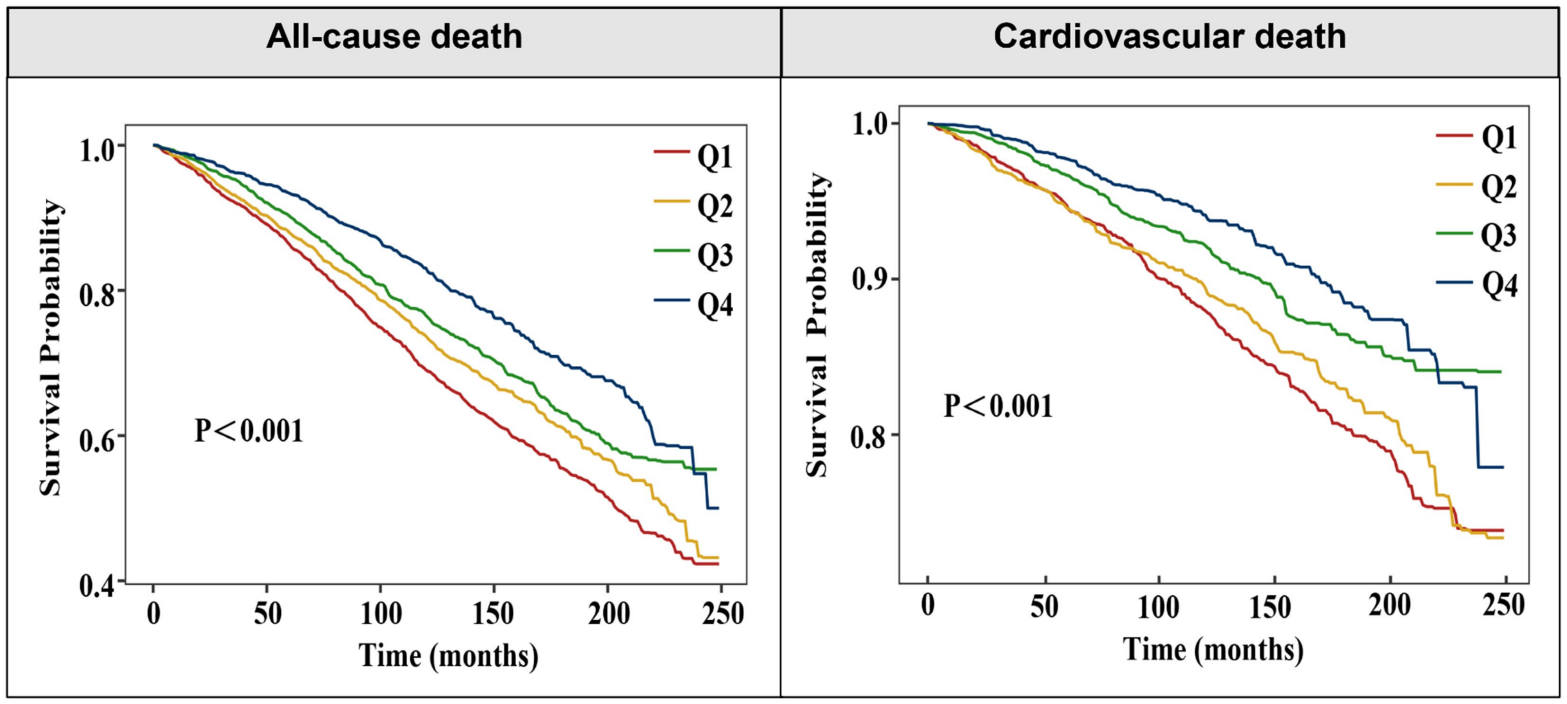
Hazard ratios for the all-cause, and cardiovascular mortality based on restricted cubic spine function for OBS levels.

To further clarify the association between OBS levels and the risk of all-cause and cardiovascular mortality, OBS was analyzed as a categorical variable in both univariate and multivariate models. After adjusting for confounders, higher OBS levels were independently linked to a diminished risk of all-cause mortality [model 3: Q2, Q3, Q4: aHR (95CI%) = 0.85 (0.75–0.96), 0.87 (0.77–0.98), 0.74 (0.62–0.88), respectively; P for trend<0.001]. An inverse association was found between elevated OBS quartiles and cardiovascular mortality, while lower SIRI quartiles showed no statistically significant relationship with cardiovascular death [model 3: Q2, Q3, Q4: aHR (95CI%) = 0.96 (0.77–1.19), 0.78 (0.63–0.97), 0.70 (0.53–0.93), respectively; P for trend = 0.003] (Table 2). Consistently, Kaplan–Meier survival curves also indicated a significantly higher survival probability associated with higher OBS levels (Figure 2). Overall, a linear inverse association between OBS levels and all-cause as well as cardiovascular mortality was supported by these findings.

### Sensitivity and stratified analyses

We conducted a sensitivity analysis by removing participants who died within 2 years of follow-up. The findings indicated that the association of OBS level with all-cause and cardiovascular mortality remained significant (Supplementary Table 2). To further explore heterogeneity in subgroup analyses, stratified analyses were performed according to age, CVD, CKD, and DM status. The findings were consistent across subgroup analyses (non-DM, CVD, and regardless of age, as well as CKD) for all-cause mortality. A similar trend in cardiovascular mortality was observed across OBS quartiles in subgroups (age≥65, non-CKD, non-DM, and CVD). Interactive analysis revealed no significant interactions between all variables for both all-cause and cardiovascular mortality (Supplementary Figures 3, 4).

Furthermore, a higher OBS was significantly associated with a lower risk of both all-cause and cardiovascular mortality in patients with only one CRM condition. In contrast, in patients with two or three co-existing CRM conditions, the inverse association between OBS and mortality was attenuated and did not reach statistical significance. These findings suggest that the protective effect of a higher OBS may be more evident in patients with a less complex disease burden (Supplementary Table 3).

## Discussion

To our knowledge, this is the first prospective research that explored the correlation between OBS level and long-term prognosis among CRM patients. Our research showed a significant reduction in all-cause mortality with higher OBS levels in a dose-dependent manner, while there was an obviously reduced risk of cardiovascular death when OBS was at a relatively high level. These findings suggest that a favorable oxidative balance is associated with lower mortality risk in this high-risk population.

Overall, oxidative homeostasis cannot be accurately assessed by a single oxidative stress factor, as multiple factors influence oxidative stress levels in the body (9). OBS, a new and composite oxidative stress indicator, gives a more complete assessment of an individual’s oxidative and antioxidant status (10). Previous studies have found that the OBS level is strongly related to mortality in patients with chronic diseases. Lan et al. found elevated OBS level was negatively related to all-cause mortality risk in adult participants with CKD (11). Luo et al. found that elevated OBS levels were related to reduced risk of mortality from both all-causes and respiratory diseases among chronic inflammatory airway diseases (12). In addition, Xu et al. also demonstrated that an inverse correlation was observed between OBS and the all-cause mortality among patients with metabolic syndrome (20). Consistent with these findings, our research revealed that higher OBS levels were associated with a decreased risk of total and cardiovascular mortality in CRM patients. Therefore, monitoring OBS levels may be helpful to assess the mortality risk in this high-risk population.

Our subgroup analysis demonstrated that higher OBS levels were negatively associated with cardiovascular mortality in both non-diabetic and non-CKD groups, but not significantly related to patients with diabetic and CKD. This discrepancy may be attributed to several potential factors. Firstly, patients with diabetes and CKD often experience a persistent state of heightened oxidative stress due to chronic inflammation, hyperglycemia, and accumulation of uremic toxins, which may blunt the protective effect of oxidative balance even when the OBS level is elevated (21). In addition, pharmacological treatments such as insulin and RAAS inhibitors commonly used in these populations may interfere with redox homeostasis, thereby attenuating the cardiovascular benefits of OBS, which might overshadow the impact of oxidative stress in statistical models (22, 23). It is noteworthy that our study defined CRM disease as a constellation of conditions that includes CVD, CKD, as well as DM. This broader inclusion may capture a more heterogeneous population, including patients in earlier stages of disease progression or those with only mild organ dysfunction (14). In these patients, oxidative balance may still play a relatively modifiable and meaningful role in influencing outcomes. Our further subgroup analysis also indicates that the protective effect of OBS may be more pronounced in patients with less complex disease clusters and diminishes in those with more advanced or multiple coexisting CRM conditions. This pattern likely reflects a threshold effect of oxidative stress, wherein advanced multi-organ dysfunction may exceed the compensatory capacity of antioxidant defenses (11, 24). Consequently, in end-stage disease states, systemic oxidative balance becomes less modifiable. In such scenarios, the cumulative oxidative damage and disease burden may overshadow the impact of oxidative balance.

The pathophysiology of the CRM condition involves a complex interplay of mechanisms, with oxidative stress serving as a key driver of disease progression. A hyperglycemic state, coupled with impaired cardiac and renal function, can simultaneously trigger the activation of the renin-angiotensin-aldosterone system and elevate levels of ROS, late glycosylation end products, and protein kinase C. Consequently, these alterations further intensify oxidative stress, leading to worsening organ damage and dysfunction (25). Furthermore, oxidative stress plays a crucial role in endothelial dysfunction, chronic inflammation, and mitochondrial impairment, all of which significantly contribute to the advancement of CRM (26, 27). In contrast, a higher OBS, characterized by increased antioxidant intake and reduced exposure to pro-oxidants, may help counteract these detrimental effects. Specifically, a higher OBS is associated with lower oxidative damage and better vascular function, thereby preserving endothelial integrity and mitigating disease progression (28). These mechanisms collectively contribute to a lower risk of mortality in CRM patients with an elevated level of OBS.

Our findings underscore the potential value of integrating oxidative balance assessment into the CRM for diseases. Since OBS is largely influenced by modifiable lifestyle factors, healthcare providers could implement targeted interventions to enhance dietary antioxidant intake while simultaneously reducing exposure to pro-oxidants (10). Furthermore, personalized nutrition plans and lifestyle modifications, such as increasing the consumption of fruits, vegetables, and polyphenol-rich foods while minimizing smoking and excessive alcohol intake, may play a crucial role in lowering mortality risk among CRM patients (29). Moreover, assessing OBS level not only provides a valuable tool for risk stratification but also deepens our understanding of the pathophysiological mechanisms underlying CRM diseases, which could offer novel insights into more effective treatment and prevention strategies in comprehensive disease management.

Although we adjusted for a wide range of demographic, clinical, and lifestyle variables, the possibility of residual confounding from unmeasured factors such as genetic susceptibility, socioeconomic status, or inflammatory markers not captured by the SII cannot be entirely excluded. To mitigate concerns regarding reverse causality, we conducted a sensitivity analysis excluding participants who died within 2 years of follow-up; the associations remained robust, suggesting that reverse causation is unlikely to fully explain our findings. Additionally, pharmacologic treatments frequently used in CRM populations, such as statins and RAAS inhibitors, may independently modulate oxidative balance through antioxidant mechanisms, while poor medication adherence could enhance oxidative stress (23). While medication use was adjusted for in our models, residual confounding or effect modification cannot be entirely ruled out. In addition, irreversible oxidative damage may further diminish the influence of modifiable antioxidant exposures in patients with more advanced disease, potentially attenuating the observed effects of the OBS (21, 24). Therefore, future studies should incorporate longitudinal assessments of OBS to better capture temporal changes in oxidative balance and establish causality (30). Moreover, incorporating the assessment of medication adherence, disease progression markers, and direct oxidative stress biomarkers could refine mechanistic insights and facilitate the design of personalized, widely applicable preventive interventions.

### Limitation

Despite its strengths, this study has several limitations. Firstly, OBS was assessed solely at baseline, which limits our ability to evaluate changes or fluctuations over time, and consequently, the need for longitudinal studies with repeated measures of oxidative balance to better establish temporality and causal relationships. Secondly, OBS was based on self-reported dietary and lifestyle data, which may be prone to recall bias. Thirdly, the study’s observational nature restricts causal conclusions, and residual confounding from unmeasured variables remains a possibility. Fourth, NHANES data do not include direct biomarkers of oxidative stress, such as ROS levels or antioxidant enzyme activity, which could further validate the relationship between OBS and mortality. Moreover, the absence of information on genetic susceptibility and medication adherence may further constrain the ability to fully elucidate individual variability in oxidative balance and its impact on clinical outcomes. Finally, since the NHANES study was conducted exclusively in the United States, the applicability of our findings to other regions remains uncertain. Therefore, further high-quality research in different populations is required to confirm these findings.

## Conclusion

Our research demonstrated a significant negative correlation between higher OBS levels and all-cause and cardiovascular mortality among CRM patients. These findings demonstrate that better oxidative balance may be related to a reduced risk of mortality, highlighting that assessing oxidative stress status may be useful for mortality risk stratification in CRM patients.

## Data Availability

Publicly available datasets were analyzed in this study. This data can be found here: https://wwwn.cdc.gov/nchs/nhanes/.

## Footnotes

^1^ https://www.cdc.gov/nchs/data-linkage/mortality-public.htm
