# Supplementary material for: Association of oxidative balance score with all-cause and cardiovascular mortality among patients with cardio-renal-metabolic disease
Source: Front Nutr. 2025 Jun 25;12:1618184. doi: 10.3389/fnut.2025.1618184 (PMC12238758; doi:10.3389/fnut.2025.1618184)
Supplement: Supplementary file 1 [file Data_Sheet_1.docx]

**Supplementary Table 1** Oxidative balance score assignment scheme

| OBS components | Property | Male | | | Female | | |
| --- | --- | --- | --- | --- | --- | --- | --- |
|  |  | 0 | 1 | 2 | 0 | 1 | 2 |
| **Dietary OBS components** | | | | | | | |
| Dietary fiber (g/d) | antioxidant | <12.56 | 12.56-19.70 | ≥19.70 | <10.10 | 10.10-16.31 | ≥16.31 |
| Carotene (RE/d) | antioxidant | <98.83 | 98.83-306.25 | ≥306.25 | <98.08 | 98.08-383.50 | ≥383.50 |
| Riboflavin (mg/d) | antioxidant | <1.79 | 1.79-2.69 | ≥2.69 | <1.34 | 1.34-2.02 | ≥2.02 |
| Niacin (mg/d) | antioxidant | <20.65 | 20.65-29.75 | ≥29.75 | <14.52 | 14.52-21.86 | ≥21.86 |
| Vitamin B₆ (mg/d) | antioxidant | <1.59 | 1.59-2.40 | ≥2.40 | <1.13 | 1.13-1.77 | ≥1.77 |
| Total folate (mcg/d) | antioxidant | <316.00 | 316.00-492.00 | ≥492.00 | <251.00 | 251.00-388.96 | ≥388.96 |
| Vitamin B₁₂ (mcg/d) | antioxidant | <3.36 | 3.36-6.20 | ≥6.20 | <2.22 | 2.22-4.22 | ≥4.22 |
| Vitamin C (mg/d) | antioxidant | <42.44 | 42.44-113.21 | ≥113.21 | <38.01 | 38.01-98.49 | ≥98.49 |
| Vitamin E (ATE) (mg/d) | antioxidant | <5.82 | 5.82-9.42 | ≥9.42 | <4.53 | 4.53-7.52 | ≥7.52 |
| Calcium (mg/d) | antioxidant | <646.00 | 646.00-1072.00 | ≥1072.00 | <499.24 | 499.24-849.00 | ≥849.00 |
| Magnesium (mg/d) | antioxidant | <257.00 | 257.00-361.28 | ≥361.28 | <187.00 | 187.00-283.43 | ≥283.43 |
| Zinc (mg/d) | antioxidant | <9.75 | 9.75-15.10 | ≥15.10 | <6.73 | 6.73-10.75 | ≥10.75 |
| Copper (mg/d) | antioxidant | <1.12 | 1.12-1.57 | ≥1.57 | <0.85 | 0.85-1.28 | ≥1.28 |
| Selenium (mcg/d) | antioxidant | <94.94 | 94.94-141.80 | ≥141.80 | <67.79 | 67.79-99.50 | ≥99.50 |
| Total fat (g/d) | prooxidant | ≥69.83 | 69.83-107.43 | <107.43 | ≥75.79 | 50.98-75.79 | <50.98 |
| Iron (mg/d) | prooxidant | ≥12.88 | 12.88-19.17 | <19.17 | ≥9.65 | 9.65-14.32 | <14.32 |
| **Lifestyle OBS components** | | | | | | | |
| Physical activity (MET-minute/week) | antioxidant | <417.86 | 417.86-1135.71 | ≥1135.71 | <270.00 | 270.00-845.71 | ≥845.71 |
| Alcohol (g/d) | prooxidant | ≥30 | 0-30 | None | ≥15 | 0-15 | None |
| Body mass index (kg/m²) | prooxidant | ≥25.54 | 25.54-29.17 | <29.17 | ≥23.74 | 23.74-28.64 | <28.64 |
| Cotinine (ng/mL) | prooxidant | ≥0.038 | 0.038-1.13 | <1.13 | ≥0.035 | 0.035-0.172 | <0.172 |

OBS: oxidative balance score; RE: retinol equivalent; ATE: alpha-tocopherol equivalent; MET: metabolic equivalent.

**Supplementary Table 2** HRs (95% CIs) of all-cause and cardiovascular mortality according to OBS levels among CRM participants after excluding participants who died within two years of follow-up

| **Groups** | **Model 1^a^** |  | **Model 2^b^** |  | **Model 3^c^** |  |
| --- | --- | --- | --- | --- | --- | --- |
|  | **HR (95%CI)** | **P Value** | **HR (95%CI)** | **P Value** | **HR (95%CI)** | **P Value** |
| **All-cause death** | | | | | | |
| **Continuous** |  |  |  |  |  |  |
| OBS | 0.98(0.97,0.98) | <0.001 | 0.98(0.97,0.99) | <0.001 | 0.98(0.97,0.99) | 0.003 |
| **Quartiles** |  |  |  |  |  |  |
| Q1 | 1.00(reference) |  | 1.00(reference) |  | 1.00(reference) |  |
| Q2 | 0.78(0.69,0.89) | <0.001 | 0.82(0.72,0.93) | 0.001 | 0.85(0.74,0.97) | 0.014 |
| Q3 | 0.76(0.68,0.85) | <0.001 | 0.81(0.72,0.91) | <0.001 | 0.87(0.76,0.98) | 0.027 |
| Q4 | 0.64(0.54,0.75) | <0.001 | 0.72(0.61,0.86) | <0.001 | 0.76(0.63,0.91) | 0.003 |
| P for trend | <0.001 |  | <0.001 |  | 0.003 |  |
| **Cardiovascular death** | | | | | | |
| **Continuous** |  |  |  |  |  |  |
| OBS | 0.97(0.96,0.98) | <0.001 | 0.98(0.97,0.99) | 0.001 | 0.98(0.97,0.99) | 0.030 |
| **Quartiles** |  |  |  |  |  |  |
| Q1 | 1.00(reference) |  | 1.00(reference) |  | 1.00(reference) |  |
| Q2 | 0.81(0.66,0.99) | 0.045 | 0.84(0.68,1.04) | 0.109 | 0.92(0.74,1.14) | 0.448 |
| Q3 | 0.70(0.57,0.86) | <0.001 | 0.73(0.60,0.90) | 0.004 | 0.82(0.65,1.02) | 0.072 |
| Q4 | 0.64(0.50,0.83) | <0.001 | 0.70(0.55,0.90) | 0.006 | 0.76(0.57,1.01) | 0.061 |
| P for trend | <0.001 |  | 0.001 |  | 0.032 |  |

^a^Model 1: adjusted for age, gender

^b^Model 2: adjusted for age, gender, race, body mass index, smoke, alcohol drinking

^c^Model 3: adjusted for age, gender, race, body mass index, smoke, alcohol drinking, hypertension, diabetes mellitus; chronic kidney disease; cardiovascular disease, hemoglobin A1c, estimated glomerular filtration rate; uric acid, blood urea nitrogen; hemoglobin; aspartate aminotransferase; systemic immune- inflammation index; total cholesterol, high-density lipoprotein cholesterol; antihypertensive drug; lipid-lowering drug.

Supplementary Table 3 Subgroups analyses for the association between OBS level and all-cause and cardiovascular mortality across the number of CRM conditions.

| **Character** | Q1 | Q2 | P-value | Q3 | P-value | Q4 | P-value | P for trend | |  |  |
| --- | --- | --- | --- | --- | --- | --- | --- | --- | --- | --- | --- |
| **All-cause mortality** | | | | | | | | |  |  |  |
| Group | | | | | | | | |  | | |
| Only CVD | reference | 0.71(0.52,0.96) | 0.031 | 0.80(0.60,1.06) | 0.122 | 0.57(0.40,0.83) | 0.003 | 0.010 | |  |  |
| Only CKD | reference | 0.83(0.64,1.08) | 0.165 | 0.73(0.57,0.93) | 0.011 | 0.77(0.60,0.99) | 0.043 | 0.013 | |  |  |
| Only DM | reference | 0.89(0.63,1.27) | 0.532 | 0.94(0.66,1.35) | 0.739 | 0.86(0.53,1.40) | 0.544 | 0.606 | |  |  |
| CVD and CKD | reference | 0.92(0.71,1.20) | 0.544 | 1.00(0.74,1.35) | 0.989 | 0.62(0.44,0.88) | 0.008 | 0.032 | |  |  |
| CVD and DM | reference | 0.51(0.32,0.82) | 0.006 | 0.49(0.29,0.85) | 0.010 | 0.68(0.29,1.56) | 0.362 | 0.224 | |  |  |
| CKD and DM | reference | 0.61(0.44,0.85) | 0.003 | 1.12(0.82,1.54) | 0.477 | 0.91(0.57,1.45) | 0.679 | 0.672 | |  |  |
| CVD and CKD and DM | reference | 1.50(1.09,2.07) | 0.013 | 1.17(0.84,1.64) | 0.359 | 0.76(0.48,1.21) | 0.252 | 0.386 | |  |  |
| The number of conditions | | | | | | | | |  | | |
| 1 | reference | 0.83(0.71,0.96) | 0.015 | 0.79(0.67,0.93) | 0.004 | 0.71(0.58,0.86) | <0.001 | <0.001 | |  |  |
| 2 | reference | 0.71(0.59,0.87) | <0.001 | 0.94(0.76,1.17) | 0.585 | 0.80(0.60,1.07) | 0.135 | 0.346 | |  |  |
| 3 | reference | 1.50(1.09,2.07) | 0.013 | 1.17(0.84,1.64) | 0.359 | 0.76(0.48,1.21) | 0.252 | 0.386 | |  |  |
| **Cardiovascular mortality** | | | | | | | | |  |  |  |
| Group | | | | | | | | |  | | |
| Only CVD | reference | 0.76(0.47,1.24) | 0.272 | 0.71(0.43,1.17) | 0.181 | 0.64(0.34,1.22) | 0.176 | 0.166 | |  |  |
| Only CKD | reference | 0.84(0.54,1.30) | 0.438 | 0.70(0.46,1.08) | 0.106 | 0.87(0.53,1.42) | 0.572 | 0.352 | |  |  |
| Only DM | reference | 0.81(0.42,1.57) | 0.532 | 0.79(0.38,1.65) | 0.527 | 0.52(0.26,1.08) | 0.078 | 0.098 | |  |  |
| CVD and CKD | reference | 1.09(0.73,1.64) | 0.681 | 1.09(0.68,1.75) | 0.718 | 0.60(0.36,1.02) | 0.057 | 0.135 | |  |  |
| CVD and DM | reference | 0.75(0.35,1.63) | 0.473 | 0.41(0.20,0.83) | 0.013 | 0.18(0.05,0.61) | 0.006 | <0.001 | |  |  |
| CKD and DM | reference | 0.75(0.42,1.33) | 0.326 | 0.94(0.56,1.58) | 0.808 | 1.40(0.63,3.10) | 0.412 | 0.395 | |  |  |
| CVD and CKD and DM | reference | 1.77(1.07,2.95) | 0.027 | 0.94(0.59,1.52) | 0.804 | 0.55(0.28,1.09) | 0.087 | 0.073 | |  |  |
| The number of conditions | | | | | | | | |  | | |
| 1 | reference | 0.85(0.65,1.11) | 0.229 | 0.73(0.54,0.98) | 0.037 | 0.70(0.49,1.00) | 0.047 | 0.028 | |  |  |
| 2 | reference | 0.89(0.64,1.24) | 0.494 | 0.87(0.63,1.19) | 0.379 | 0.80(0.52,1.22) | 0.299 | 0.251 | |  |  |
| 3 | reference | 1.77(1.07,2.95) | 0.027 | 0.94(0.59,1.52) | 0.804 | 0.55(0.28,1.09) | 0.087 | 0.073 | |  |  |

Q, Quartile; CVD, cardiovascular disease; DM, diabetes mellitus; CKD, chronic kidney disease


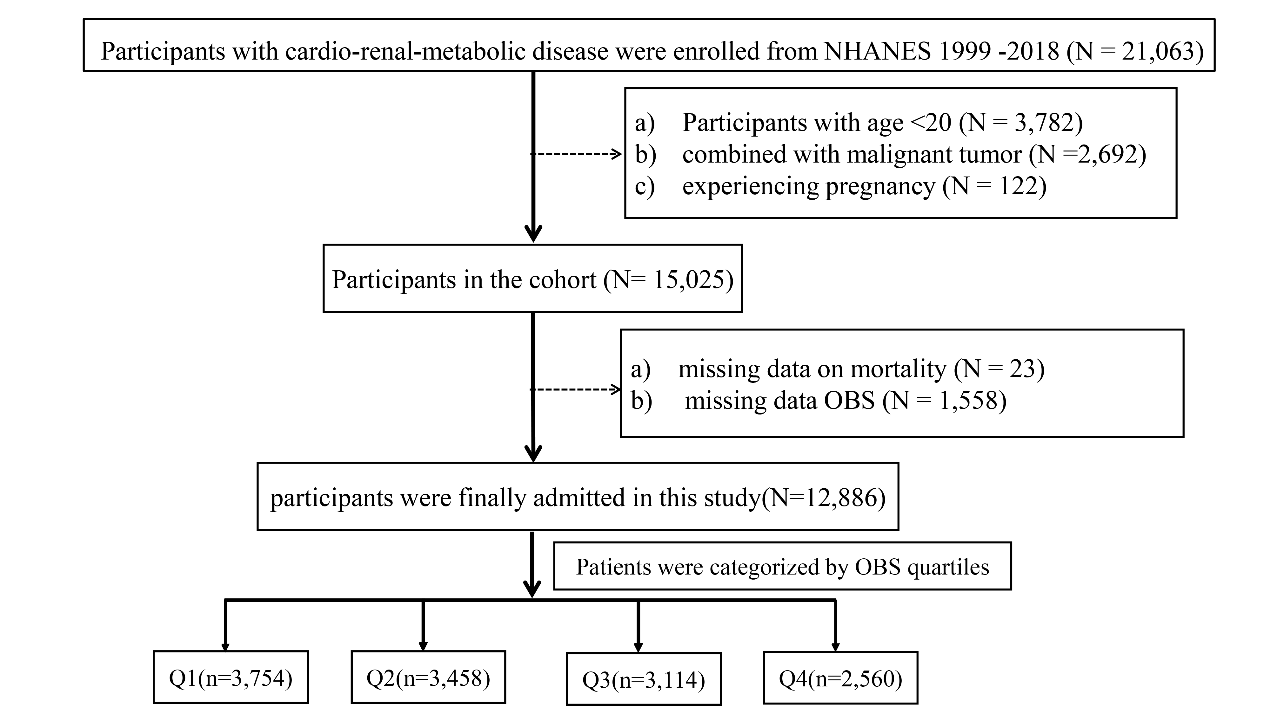


**Supplemental Figure.1** Flowchart of study participants. OBS: oxidative balance score


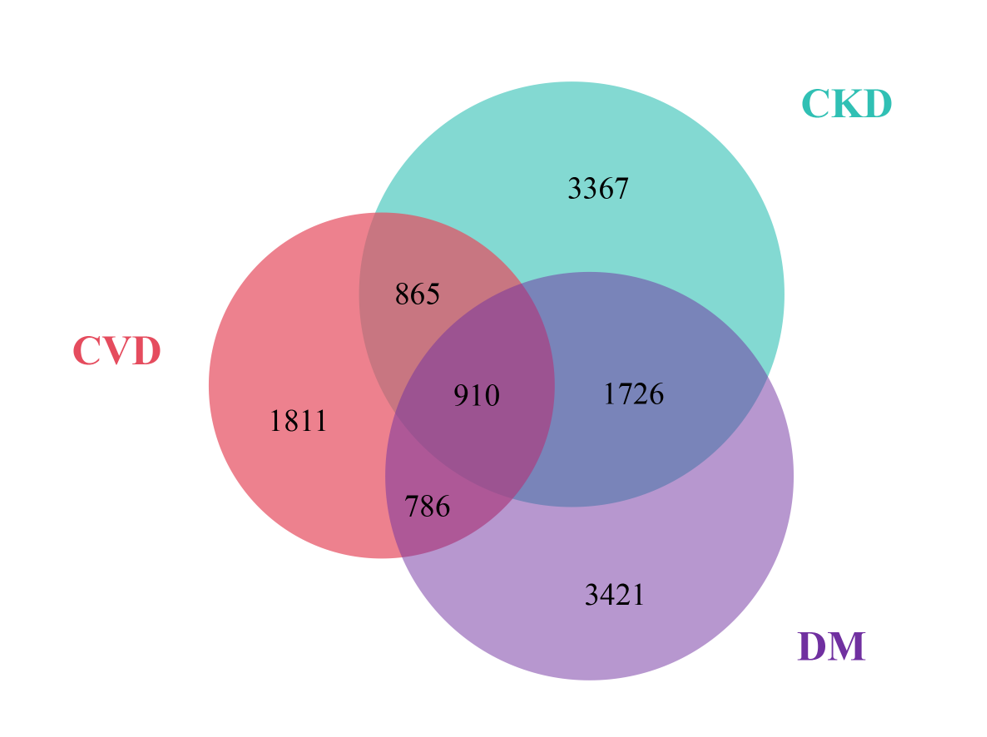


**Supplemental Figure.2** Distribution of cardio-renal-metabolic conditions in the study cohort

CVD, cardiovascular disease; DM, diabetes mellitus; CKD, chronic kidney disease


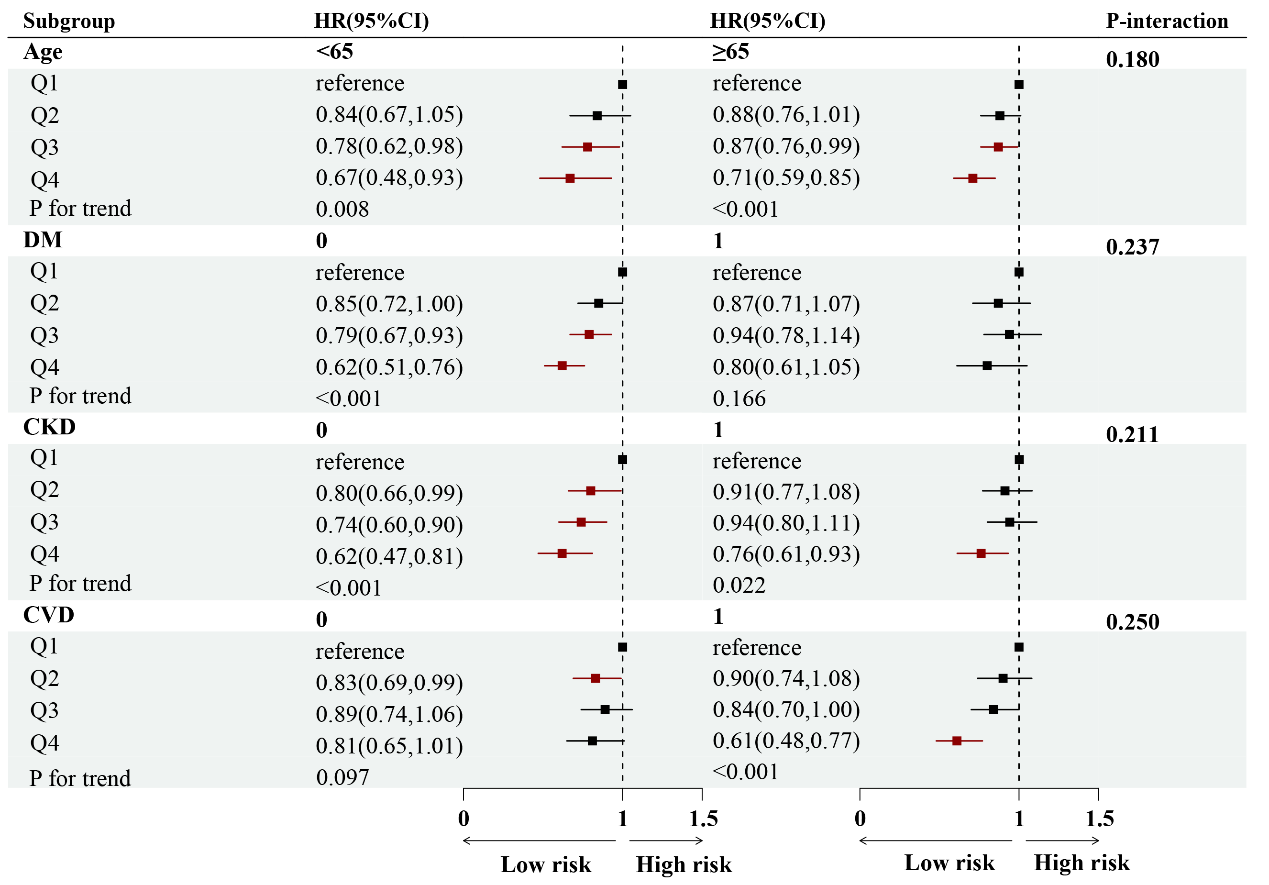


**Supplemental Figure.3** Subgroups analyses for the association between OBS level and all-cause mortality


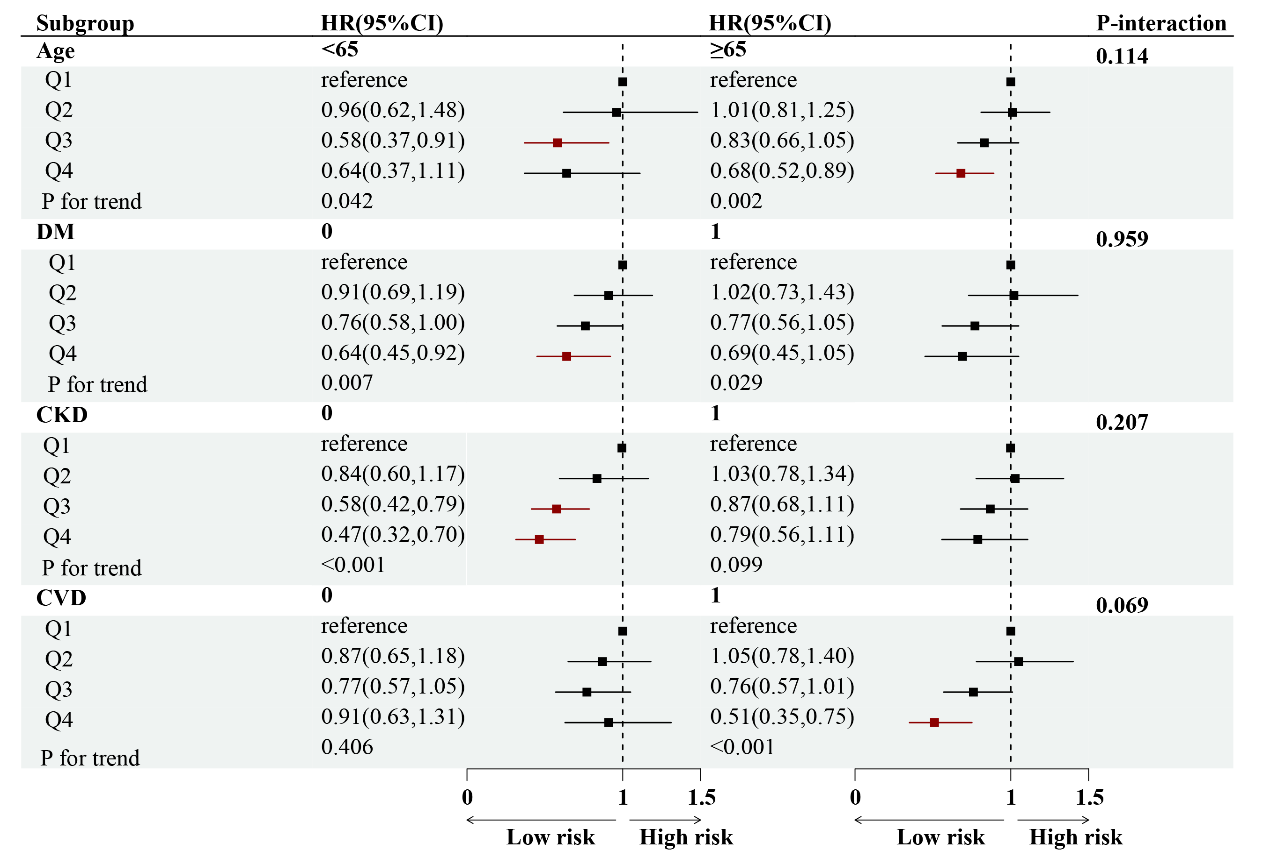


**Supplemental Figure.4** Subgroups analyses for the association between OBS level and cardiovascular mortality
